# The Weight of the Brain and Its Functional Activity

**Published:** 1881-05

**Authors:** 


### THE WEIGHT OF THE BRAIN AND ITS FUNCTIONAL ACTIVITY.

Dr. Ambrose L. Ranney, Adjunct Professor of Anatomy in the Medical Department of the University of the City of New York, contributes to the May number of the *New York Medical Journal* an article on "Some Points in the Anatomy and Physiology of the Brain, and their Practical Bearings," from which we extract the following: The shape of the cranium may indicate the relative size of the different parts of the encephalon, and the circumference of the head and the height of the skull above the orifice of the ear may also relatively indicate the measurements of the cerebrum and its basal ganglia (which are inclosed within it). The variations in the skulls of the different nations indicate an amount of brain which is in direct ratio to the facial angle of Camper. The average weight of the brain of a healthy adult of the Caucasian race has been given, by most of the prominent investigators upon this subject, as about fifty ounces in the male and some six ounces less in the female. In the new-born infant the weight of the brain in the two sexes is more nearly alike, being about eleven ounces for the mail child and ten ounces for the female. The rapidity of growth of the brain is not uniform throughout the different periods of life, since it grows rapidly until the age of seven years, then less rapidly until the age of forty is reached, when it attains its full development, and after that age it decreases in weight about one ounce for every period of ten years. The comparative weights of the component parts of the encephalon are, in approximate figures, about one fiftieth of the entire weight for the pons Varolii and the medulla oblongata taken together; one tenth of the entire weight for the cerebellum; and the balance of the total weight for the cerebrum and the basal ganglia inclosed within its substance. These proportions also show a slight variation in the two sexes, but not to so marked an extent as to render this statement far from a correct one. It may be stated, as a rule, that the relative proportion of the cerebrum to that of the cerebellum is greater in the intellectual races; and that the cerebrum is developed in individuals in proportion to their intellectual power, although the absolute size may not be taken as a guide to the quality of the mind, since it is undoubtedly true that the brain can be improved *in quality* by exercise, as well as the muscular tissue. That there are important individual differences in the quality of the generating nervous matter is evidenced by the facts that some small brains actuallyaccomplish more and better work than larger ones, and that many women often show a higher degree of mental acumen than men, in spite of the fact that their brains are lighter.

